# Comparison of Machine Learning Methods to Predict Early Mortality After Evacuation of Chronic Subdural Hematoma

**DOI:** 10.1227/neuprac.0000000000000151

**Published:** 2025-07-25

**Authors:** Trenton A. Line, Anoop S. Chinthala, Barnabas Obeng-Gyasi, Gordon Mao, Jamie L. Bradbury, Aditya Mittal, Jan Vargas, Ryan T. Kellogg, Enyinna Nwachuku, David O. Okonkwo, Matthew Pease

**Affiliations:** * Department of Neurological Surgery, Indiana University School of Medicine, Indianapolis, Indiana, USA;; ‡ Department of Neurosurgery, University of Pittsburgh Medical Center Medical School, Pittsburgh, Pennsylvania, USA;; § Division of Neurosurgery, PRISMA Health, Greenville, South Carolina, USA;; ‖ Department of Neurosurgery, University of Virginia, Charlottesville, Virginia, USA;; ¶ Department of Neurosurgery, Cleveland Clinic Foundation, Cleveland, Ohio, USA;; # Department of Neurosurgery, UPMC Healthcare System, Pittsburgh, Pennsylvania, USA

**Keywords:** Artificial intelligence, Chronic subdural hematoma, Machine learning, Mortality, Predictive modeling

## Abstract

**BACKGROUND AND OBJECTIVES:**

We developed a series of machine learning models to predict early mortality after chronic subdural hematoma (cSDH) evacuation.

**METHODS:**

We retrospectively collected patients treated surgically for cSDH at 4 level 1 trauma centers (2009-2021). Previously, we developed a deep learning segmentation tool to automatically calculate preoperative and postoperative cSDH volumes. Using cSDH volumes and clinical information, we developed 6 machine learning models including logistic regression (LR), support vector machine, neural network (NN), decision tree (DT), Naïve Bayes, and XGBoost to predict 30-day mortality after surgery. We applied least absolute shrinkage and selection operator regression to select a subset of predictors for consistent model input. To account for class imbalance, we used synthetic minority oversampling technique. We used 10-fold cross validation to evaluate model performance.

**RESULTS:**

We included 731 patients. Our final models included age, admission Glasgow Coma Scale, unilateral/bilateral hematoma, antiplatelet status, platelet count, preoperative volume, and method of surgical evacuation. The 30-day mortality rate was 7.5%. Overall, our models demonstrated moderate discriminative ability with area under the receiver operating characteristics curves (AUCs) ranging from 0.64 for DT (95% CI: 0.56-0.72) to 0.75 for LR (95% CI: 0.69-0.81). AUC for DT was significantly lower than LR (*P* < .03). AUCs for support vector machine (AUC = 0.73; 95% CI: 0.67-0.79), NN (0.69; 95% CI: 0.62-0.76), Naïve Bayes (0.70; 95% CI: 0.63-0.78), and XGBoost (0.73; 95% CI: 0.66-0.80) were not significantly different from LR. LR achieved the highest balanced accuracy (0.69) whereas DT and NN had the lowest (0.61). Age, craniotomy, Glasgow Coma Scale, larger preoperative volumes, unilateral cSDH, and lower platelet count were associated with increased risk of mortality on multivariate analysis.

**CONCLUSION:**

The LR model demonstrated the best performance of discriminative ability, balanced accuracy, and recall, whereas DT modeling performed worst. Using an automated segmentation software, our models demonstrate an ability to identify patients at high risk of mortality after treatment for cSDH.

**ABBREVIATIONS:**
cSDH: chronic SDH
DT: decision tree
LASSO: least absolute shrinkage and selection operator
LR: logistic regression
ML: machine learning
NB: Naïve Bayes
NN: neural network
SDH: subdural hematomas
SMOTE: Synthetic Minority Oversampling Technique
SVM: support vector machine
XGB: XGBoost.

**CODE:** https://github.iu.edu/treline/cSDH/tree/Neurosurgery-Practice

**CODE FUNCTIONALITY:** Binary classification

**DATASETS:** Proprietary. The availability of data used in this study is subject to restrictions. Data will be made available upon request to the corresponding author.

**KEY RESULTS:**

**Table:** 

Outcome	Best model	AUC (95% CI)	Recall	Balanced accuracy
30-day mortality	Logistic Regression	0.75 (0.69-0.81)	0.67	0.69

**Task:** Binary classification for predicting early mortality after cSDH evacuation

**Dataset:** Proprietary

**Best Model Name:** Logistic Regression

**Metric Name:** Area under the ROC curve

**Metric Score:** 0.75 (95% CI: 0.69-0.81)

**Metric Name:** Balanced Accuracy

**Metric Score:** 0.69

**Metric Name:** Recall

**Metric Score:** 0.67

Chronic subdural hematomas (cSDHs) are among the most commonly treated neurosurgical pathologies with an incidence estimated between 8 and 20 per 100 000 person-years.^1^ By 2030, cSDH is projected to become the most commonly treated neurosurgical pathology, with over 60 000 new cases each year.^2^ Exacerbated by high recurrence rates and mortality, cSDH presents a significant public health burden.^3,4^ Outcomes after cSDH evacuation remain suboptimal despite advancements in surgical treatment. Nearly 20% of patients have poor long-term functional outcomes. Perioperative mortality rates are approximately 10% overall and approach 30% in the elderly.^5^

Although several studies have previously identified patients at high risk of cSDH recurrence, very little literature exists to identify patients at high risk of mortality.^1,6–9^ The development of accurate models predicting mortality would allow for improved patient counseling and avoidance of futile surgeries in higher risk patients. Machine learning (ML) has emerged as a transformative tool in predictive analytics in medicine.^10^ ML provides potential to improve predictive models for mortality in cSDH, addressing limitations in current diagnostic and prognostic approaches.^11^ ML models have been developed to determine clinical and radiological factors for poor outcomes in cSDH, which can aid surgeons in recognizing high-risk patients.^12^ Recent efforts have focused on constructing models that not only predict postoperative recurrence but also identify patients who may not need surgery.^8,13-15^ Although ML models tend to perform best when trained on large data sets, advances in ML technology have facilitated the identification of key variables in smaller data sets through innovative sampling techniques, increasing the range of their potential applications.^16^

The objective of this study was to develop ML models to identify patients at high risk of mortality after cSDH intervention and evaluate the performance of various ML models on cSDH mortality. We incorporated cSDH volume using a previously developed deep learning segmentation model to calculate preoperative and postoperative volumes.^17^ We trained several common ML methods to perform a binary classification task on a large multicenter data set and then compared model performance for prediction of mortality after surgical intervention for cSDH. We hypothesized that the more complex ML methods would perform better than the logistic regression (LR) model.

## METHODS

### Ethics Approval

This study received approval from the Human Research Protection Office at all sites without need for consent.

### Data Availability

The availability of data used in this study is subject to restrictions. Data will be made available upon request to the corresponding author.

### Guidelines

We adhered to the Standards for Reporting of Diagnostic Accuracy Studies.^18^

### Patient Selection and Data Collection

We collected retrospective data from 4 tertiary, level 1 trauma centers at 3 institutions from February 2009 to August 2021. We searched the medical records using Common Procedural Technology codes for craniotomy and/or burr-hole evacuation for subdural hematoma and identified all of those treated surgically for cSDH. We reviewed patient charts for demographic data, baseline radiographical and clinical characteristics, and outcomes. We selected 30-day mortality given its relevance to perioperative risk assessments and its potential to inform postoperative resource allocation, care transitions, and discharge planning. We defined 30-day mortality from the time of surgical evacuation. We excluded patients with incomplete records or were missing surgery data. No patients underwent middle meningeal artery embolization.

As previously described, we developed an automatic cSDH volume segmentation software. We used this previously developed software to segment and calculate cSDH volumes using a preoperative and postoperative computed tomography (CT) head scan.^17^ All postoperative head CTs were obtained within 48 hours of surgery. We excluded patients missing preoperative and postoperative CT head volumes. For bilateral cSDH, we defined the preoperative and postoperative SDH volumes as the sum of the volumes from each side.

### Supervised ML Model Development

Imputation for missing data was performed using predictive mean matching. Continuous predictors were normalized by centering and scaling around their means and verified to meet model assumptions before analysis. A fixed seed was set for cross validation and model implementation to ensure consistency in training and test sets between models. Categorical predictors were converted to binary features using one-hot encoding.

We developed 6 supervised ML algorithms to predict thirty-day mortality, including LR, support vector machine (SVM), neural network (NN), decision tree (DT), Naïve Bayes (NB), and XGBoost (XGB). We selected these models and methods following previous work by Arefan et al,^19^ who compared the performance of LR, SVM, NN, DT, and NB in predicting mortality after traumatic brain injuries. These models are common in clinical applications because of their ease of interpretability, computational efficiency, and suitability for tabular data. SVM and NN are less easily interpreted but are capable of capturing more complex relationships, providing a comparison across a spectrum of model complexity and interpretability. Given the inferior performance of DT in Arefan et al,^19^ we included XGB, a more sophisticated tree-based ensemble method, for comparison. All models were trained and evaluated using 10-fold stratified cross-validation. Stratified cross validation recursively splits the data into separate training folds and a test fold of equal distribution. This method works to optimize training by mitigating overfitting and underfitting while simultaneously testing the model's generalizability.^13^ To address class imbalance (mortality is a significant minority class) in the data set, we used the Synthetic Minority Oversampling Technique (SMOTE) during cross-validation in the training folds only. SMOTE generates synthetic samples for the minority class by interpolating between existing minority class observations and their nearest neighbors.^20^ This approach ensures a balanced class distribution within each training fold, mitigating bias in model training. SMOTE parameters were set to achieve a minority-majority class ratio of 1 in the training folds using 5 nearest neighbors for imputation. All models, except LR, underwent hyperparameter tuning on validation folds through a grid search or automatic selection. A 10-fold cross-validated least absolute shrinkage and selection operator (LASSO) technique was used to select a subset of predictors that minimized binomial deviance on the training sets as input for prediction models.

The following describes how models were implemented:

1. LR: We used a generalized linear model with an intercept and linear term for each predictor.
2. SVM: We used a radial basis function kernel and grid search for hyperparameter tuning to select the regularization parameter and kernel scale.
3. NN: We used a feedforward NN with a single hidden layer with 10 hidden neurons, 1000 maximum epochs, and grid search tuning for the decay parameter to prevent overfitting. Training optimization used a backpropagation algorithm with gradient descent.
4. DT: We used a classification and regression tree algorithm for recursive partitioning and used *caret* built in grid search to automatically select from 10 values to optimize the complexity parameter.
5. NB: We used a grid search for hyperparameter tuning to select LaPlace smoothing parameter, kernel density estimation, and kernel width adjustment.
6. XGB: We used a tree-based gradient boosting approach with a binary logistic loss function and used *caret* built in grid search to automatically select from 10 values for number of boosting iterations, maximum depth, *eta*, *gamma*, column sample by tree fraction, minimum child weight, and subsample fraction.

### Statistical Analysis of Model Performance

Model performance was evaluated using the area under the receiver operating characteristics curve (AUC), balanced accuracy, and recall, derived from the predictions in each test fold of cross-validation. Recall is equivalent to sensitivity or the true-positive rate. Balanced accuracy, calculated by taking the average of sensitivity and specificity, is a more useful measure of performance than accuracy in imbalanced data sets.^21^ Receiver operating characteristic curves were plotted for model comparison. AUC CIs (95%) were calculated using bootstrap methods. Cross-validated paired *t*-tests evaluated differences in AUCs between LR and other models and the Benjamini-Hochberg procedure corrected for false discovery rate.^22^ We used LR as the reference as it is the most commonly used of the methods. We performed the Shapley Additive Explanations approach on the best performing model to identify the relative importance of each predictor.^23^ All coding was performed in RStudio 2024.09.0+375 (RStudio, PBC). Models were developed using the caret, nnet, naivebayes, and XGBoost packages.^24-27^

## RESULTS

**FIGURE 1.**
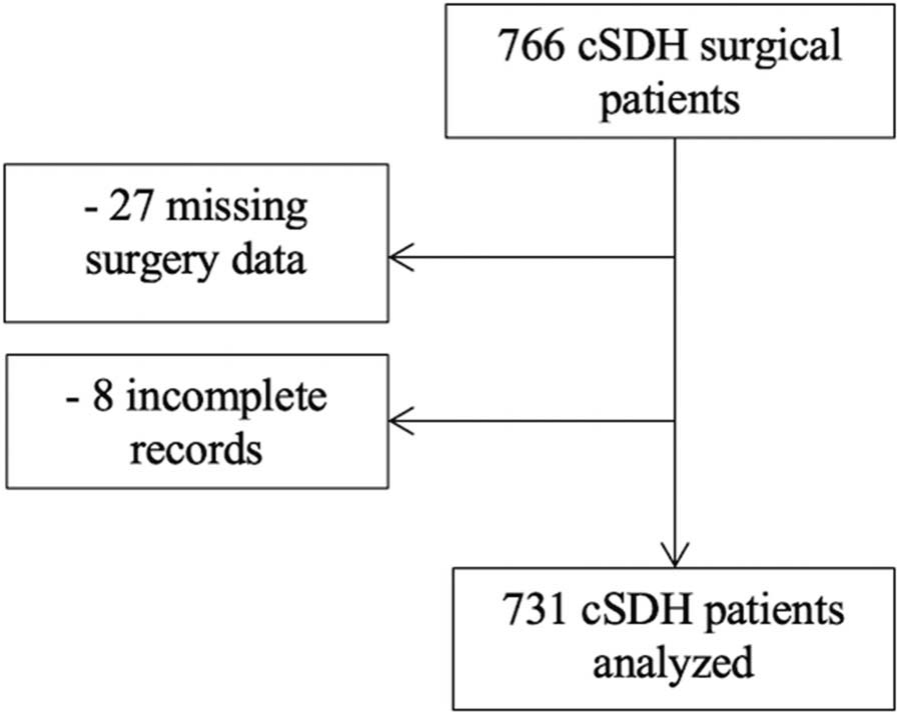
Consolidated Standards of Reporting Trials (CONSORT) diagram for our cohort. cSDH, chronic subdural hematoma.

We initially identified 766 patients treated surgically for cSDH. We excluded 27 patients with missing surgery data and 8 with incomplete records (Figure 1). We included a total of 731 patients for analysis. Table 1 displays the demographics, clinical characteristics, and rate of 30-day mortality for the cohort. The mortality rate was 7.5% overall. The median age was 75 years, and 23% had bilateral cSDH. We stratified each characteristic by 30-day mortality and calculated the *P*-values using χ^2^ and Wilcoxon rank sum tests. The 30-day mortality group was significantly older (*P* < .001), had lower admission Glasgow Coma Scale (GCS, *P* < .001), had larger preoperative cSDH volumes (*P* = .01), and underwent a higher proportion of craniotomies (*P* < .01) compared with the no mortality group (Table 1).

**TABLE 1.** Patient Demographics and Characteristics Stratified by 30-d Mortality

Characteristic	Total (n = 731^a^)	Mortality (n = 55^a^)	No mortality (n = 676^a^)	*P*-value
Female	208 (28%)	14 (25%)	194 (29%)	.6
Age (y)	75 (68, 82)	80 (75, 87)	74 (65, 81)	<.001***
Admission GCS	15 (14, 15)	14 (13, 15)	15 (14, 15)	<.001***
Bilateral cSDH (vs unilateral)	168 (23%)	10 (18%)	158 (23%)	.4
Anticoagulation	126 (17%)	9 (16%)	117 (17%)	.9
Antiplatelets	333 (46%)	31 (56%)	302 (45%)	.09
Platelet count (×10^9^/mL)	221 (177, 273)	213 (158, 266)	222 (179, 273)	.09
Preoperative cSDH volume (mL)	115 (83, 155)	131 (103, 175)	114 (82, 152)	.01*
Midline shift (mm)	7 (4, 11)	7 (4, 10)	7 (4, 11)	.4
Postoperative cSDH volume (mL)	47 (28, 75)	51 (35, 84)	46 (28, 74)	.07
Surgery type				<.01**
Burr hole evacuation	393 (54%)	20 (36%)	373 (55%)	
Craniotomy	338 (46%)	35 (64%)	303 (45%)	
30-d mortality	55 (7.5%)	—	—	—

cSDH, chronic subdural hematoma; GCS, Glasgow Coma Scale. * *P* <.05; ** *P* <.01; *** *P* <.001.

a n (% of strata); median (Q1, Q2); *P*-values calculated using χ^2^ tests (female, anticoagulation, antiplatelets, craniotomy, surgery type) and Wilcoxon rank sum tests (age, GCS, platelet count, preoperative cSDH volume, postoperative cSDH volume, midline shift).

### Model Performance

**FIGURE 2.**
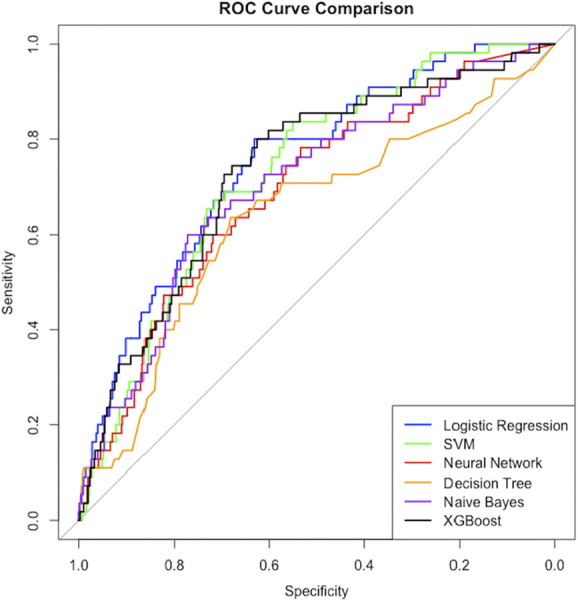
ROC curves for mortality predictions from 10-fold cross validation. ROC, receiver operating characteristic curve; SVM, support vector machine.

AUC values for the models ranged from 0.64 to 0.75 for predictions of 30-day mortality. LR demonstrated the highest AUC (0.75; 95% CI: 0.69-0.81), recall (0.67), and balanced accuracy (0.69). Decision tree performed the worst out of all models with a significantly lower AUC compared with LR (mean difference = 0.13; 95% CI: 0.04-0.22, *P* = .03). Discriminative ability for SVM, NN, NB, and XGB did not differ significantly from LR. The only models with recall above 50% were LR (0.67) and NB (0.64). Table 2 displays the performance results from cross validation for each model, and Figure 2 displays a comparison of the receiver operating characteristic curves. Table 3 displays the variables selected by LASSO for model input. **Supplemental Digital Content 1** (http://links.lww.com/NEU/E908) displays final model hyperparameters.

**TABLE 2.** Performance of Machine Learning Models Predictions From 10-Fold Cross Validation

Model	AUC	CI	MD (CI)	*P*-value^a^	Recall	ACC
Logistic regression	0.75	0.69-0.81	REF	REF	0.67	0.69
Support vector machine	0.73	0.67-0.79	0.02 (−0.04 to 0.08)	.40	0.47	0.63
Neural network	0.69	0.62-0.76	0.06 (−0.02 to 0.13)	.21	0.34	0.61
Decision tree	0.64	0.56-0.72	0.13 (0.04 to 0.22)	.03*	0.40	0.61
Naïve Bayes	0.70	0.63-0.78	0.04 (−0.02 to 0.10)	.21	0.64	0.68
XGBoost	0.73	0.66-0.80	0.01 (−0.01 to 0.04)	.26	0.45	0.63

ACC, balanced accuracy; AUC, area under the receiver operating characteristics curve; MD, mean difference in AUC across validation folds compared to logistic regression; REF, reference model for cross-validated paired *t*-test.

a *P*-values corrected for multiple comparisons.

**TABLE 3.** Variables Selected for Model Input Assessed Using 10-Fold Cross-Validated LASSO Technique

Characteristic	Included
Sex (Female)	—
Age	Y
Admission GCS	Y
Bilateral cSDH (vs unilateral)	Y
Anticoagulation	—
Antiplatelets	Y
Platelet count	Y
Preoperative cSDH volume	Y
Postoperative cSDH volume	—
Midline shift	—
Craniotomy (vs burr hole)	Y

cSDH, chronic subdural hematoma; GCS, Glasgow Coma Scale; LASSO, least absolute shrinkage and selection operator.

Y = Included.

**FIGURE 3.**
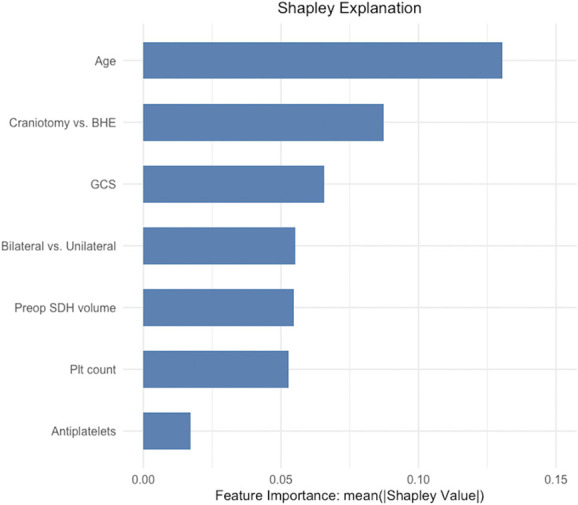
SHAP feature importance of LR model features. This figure displays the relative importance of each input feature for the LR model predictions, calculated using the SHAP method. Features with higher mean absolute Shapley values contributed more to the model's decisions. BHE, burr-hole evacuation; GCS, Glasgow Coma Scale; LR, logistic regression; Plt, Platelet; SDH, subdural hematoma; SHAP, Shapley Additive Explanations.

The relative global feature importance, calculated using the Shapley Additive Explanations method, was highest for age, followed by method of surgical evacuation and admission GCS, indicating that these features contributed most to the LR model's predictions (Figure 3). We extracted the beta coefficients and standardized odds ratios (OR) for the LR model, presented in Table 4. Bilateral hematoma (β = −0.95; OR = 0.39, 95% CI: 0.26-0.57, *P* < .001), platelet count (β = −0.44; OR = 0.64, 95% CI: 0.54-0.75, *P* < .001), and admission GCS (β = −0.39; OR = 0.68, 95% CI: 0.62-0.73, *P* < .001) had significant negative coefficients. Craniotomy (β = 0.99; OR = 2.67, 95% CI: 2.05-3.55, *P* < .001), age (β = 0.98; OR = 2.66, 95% CI: 2.24-3.17, *P* < .001), and preoperative volume (β = 0.40; OR = 1.49, 95% CI: 1.28-1.73, *P* < .001) had significant positive coefficients. A variance inflation factor test confirmed a lack of multicollinearity among LR model predictors.

**TABLE 4.** Beta Coefficients and Standardized ORs for the Logistic Regression Model Input Features

Predictor	Beta coefficient	OR^a^ (95% CI)	*P*-value
Age	0.98	2.66 (2.24-3.17)	<.001
Preoperative cSDH volume	0.40	1.49 (1.28-1.73)	<.001
Craniotomy	0.99	2.69 (2.05-3.55)	<.001
Antiplatelets	0.20	1.22 (0.92-1.61)	.16
Bilateral cSDH	−0.95	0.39 (0.26-0.57)	<.001
Platelet count	−0.44	0.64 (0.54-0.75)	<.001
Admission GCS	−0.39	0.68 (0.62-0.73)	<.001

cSDH, chronic subdural hematoma; GCS, Glasgow Coma Scale; OR, odds ratio.

a Standardized odds ratios.

We calculated the spearman correlation coefficients to further display the relationships between each predictor and mortality in our data set. No predictors had a strong correlation with the outcome. Only GCS (ρ = −0.19), age (0.16), craniotomy (0.10), and preoperative cSDH volume (0.09) had weak relationships with mortality. Antiplatelets (ρ = 0.06), bilateral cSDH (−0.03), and platelet count (−0.06) were not correlated with mortality.

## DISCUSSION

In this study, we trained 6 supervised ML models to predict 30-day mortality after surgical evacuation of cSDH in a multi-institutional, retrospective cohort of 731 patients. We used an automated, deep learning cSDH segmentation software to account for cSDH volumes. Despite the highly imbalanced outcome distribution, several models achieved moderate discriminative performance. LR outperformed all other models of AUC (0.75), balanced accuracy (0.69), and recall (0.67). Decision tree performed the poorest with a significantly lower AUC compared with LR (mean difference = 0.13; 95% CI: 0.04-0.22, *P* = .03). AUCs for the more complex models—support vector machine, NN, NB, and XGB—were not significantly different from the LR model, but only the LR and NB models achieved true-positive rates above 50%.

Our study has several strengths. First, we developed several models to identify patients at high risk of early mortality after cSDH. Consistent with previous literature, patients undergoing craniotomy had higher rates of mortality.^28^ Patients with older age, lower admission GCS, higher preoperative volumes, and lower platelet counts were also at increased risk of mortality.

Second, our study applied supervised ML models to predict mortality in cSDH patients. Similar ML models have shown success in predicting postoperative recurrence, reoperation, hematoma self-resorption, and functional outcomes in cSDH patients, but very little literature exists to predict mortality.^8,12-15,29^ Several models trained to predict recurrence were successful despite significant class imbalance.^13,29^ In an approach similar to this study, Ni et al used LASSO for variable selection and SMOTE for class balancing and trained XGB, LR, and SVM models to predict recurrence (15.1% recurrence rate). In line with our results, LR (AUC = 0.71; recall = 73%) outperformed SVM (AUC = 0.61; recall = 47%) and both showed limited performance. However, their XGB model achieved excellent predictive performance (AUC = 0.90; recall = 89%).^29^ Fang et al trained a convolutional NN, SVM, and LR model to predict recurrence (16.7% recurrence) using clinical data combined with radiomics. In contrast to our results, SVM (AUC = 0.86; recall = 0.83) and NN (AUC = 0.84; recall = 79%) demonstrated good performance. Performance for LR was less impressive and similar to our LR model (AUC = 0.77; recall = 0.69).^13^ Aside from the impact of differences in target outcome and model training methods, the superior performance of the SVM, NN, and XGB models in these studies is likely a result of the rich data sets that were used for model training. Ni et al selected from 35 variables and used 10 for model input, including medical history and a wide variety of laboratory studies.^29^ Fang et al^13^ used 12 variables for model input including a variety of clinical, radiometric, and imaging data. The strength of complex ML models such as SVM, XGB, and convolutional NN is their ability to learn complex, high dimensional, and hidden patterns in data; training these models on small or low dimensionality data sets can lead to overfitting and poor performance.^12,29^

Third, we demonstrated that LR is a powerful predictive model type in clinical data sets. Despite the class imbalance, the LR model was able to achieve moderate predictive performance, outperforming the more complex models. Although SVM (AUC = 0.73) and XGB (AUC = 0.73) displayed similar discriminative performance to LR (AUC = 0.75), their recall rates were much lower (SVM: 0.47, XGB: 0.45 vs LR: 0.67). In training on highly imbalanced data sets, ML models can learn to ignore the minority class in its predictions. This can lead to seemingly impressive performance as measured by traditional accuracy and AUC, while the model fails altogether at identifying the outcome of interest, highlighting the importance of a multifaceted approach to model evaluation.^13^ Before adjusting the class imbalance in our model development, the models were achieving excellent accuracy but were failing to identify cases of mortality, as evidenced by balanced accuracy around 50%. To ensure that the minority class was not ignored, we used the SMOTE.^20^ SMOTE introduces synthetic datapoints for the minority class into the training data by sampling a number of similar observations, bringing the classes into balance. This method substantially improved the performance of all our models, increasing the number of true positives predicted on the unbalanced test sets. Of note, we attempted several other methods of addressing class imbalance, including class weighting, simple random oversampling and undersampling, manual hyperparameter tuning, training on precision recall curve metrics and balanced accuracy, and different combinations of these methods, but they failed to mitigate the training bias.

SMOTE is recommended for binary classification tasks performed by weak classifier models (LR, SVM, DT, NN).^30^ It is not generally recommended for strong classifiers, such as XGB, as it does not improve performance over simple oversampling.^30^ However, we aimed to use consistent training data between models, and other methods of mitigating bias did not substantially improve XGB performance. Limitations of SMOTE include risks of synthetic bias and overfitting.^31^ To mitigate these risks, we applied SMOTE only in the training folds, whereas validation was performed on the original unbalanced data. The beta coefficients and ORs extracted from the LR model should be interpreted with caution, however, since they were influenced by the synthetic data. Although SMOTE can cause overfitting, our models were previously showing signs of underfitting and bias, so a modest increase in variance was an acceptable tradeoff for improving minority class detection.

Fourth, we replicated previous work by demonstrating the inferior performance of the classification and regression tree DT compared with LR.^19^ Decision trees are prone to overfitting and do not deal well with class imbalance. XGB is a DT-based ensemble method that is less prone to overfitting and is known for its ability to handle sparse data.^27^ XGB exhibited superior discriminative ability compared with DT (AUC = 0.73 vs 0.64), although both approaches had low recall (XGB: 0.45 vs DT: 0.40), indicating neither model was useful for identifying cases of mortality.

### Limitations

This study has several limitations. The data were collected retrospectively, introducing the risk of bias in data collection. There was a risk of selection bias as data were collected only from level 1 trauma centers, limiting generalizability to other clinical settings and the broader US population. Overall model performance was modest. This could indicate an inherent difficulty in predicting mortality after cSDH, or it may result from limitations in our data set. We collected a relatively small number of patient characteristics to use as model input, and only collected variables from the preoperative and immediate postoperative period. We were unable to accurately collect important data points, such as frailty and comorbidities, due to the retrospective nature of the study and inconsistent documentation across patients and institutions. Mortality after hematoma evacuation is likely influenced by a much larger number of factors. Future studies aiming to develop prediction models should take into account frailty and other medical risk factors.^8,12,13,29^

## CONCLUSION

In a large data set across 4 institutions, we trained 6 ML models using state-of-the-art techniques to account for class imbalance to predict 30-day mortality after surgical evacuation of cSDH. We were able to identify patients at high risk of mortality, including those with older age, larger preoperative volumes, and who underwent craniotomy. We compared the predictive performance of various models and found that LR outperformed all other models. AUCs for the more complex models—support vector machine, NN, NB, and XGB—were not significantly different from the LR model.

## SUPPLEMENTAL DIGITAL CONTENT

**Supplemental Digital Content 1**. Final model hyperparameters http://links.lww.com/NS9/A58.
